# Lymph Node Metastasis and Recurrence Patterns in Clinical Stage IA Lower-Lobe Non-Small Cell Lung Cancer: Toward an Optimal Surgical Strategy for Superior Segment (S6) Tumors

**DOI:** 10.5761/atcs.oa.25-00198

**Published:** 2026-01-14

**Authors:** Souichiro Suzuki, Yuta Matsubayashi, Keiyu Sato, Osamu Noritake, Takuya Matsui, Katsutoshi Seto, Noriaki Sakakura

**Affiliations:** Department of Thoracic Surgery, Aichi Cancer Center, Nagoya, Aichi, Japan

**Keywords:** non-small cell lung cancer, superior segment, segmentectomy, lymph node dissection, lymph node metastasis, lymph node recurrence

## Abstract

**Purpose:**

The superior segment (S6) may differ from the basal segments (BSs) in lymphatic spread, affecting surgical strategy and mediastinal lymph node dissection (LND). We aimed to define lymphatic spread patterns and to guide surgical strategy in S6 non-small cell lung cancer (NSCLC).

**Methods:**

We reviewed 375 patients with cT1a–cT1c N0 lower-lobe NSCLC (S6, 168; BS, 207) who underwent segmentectomy or lobectomy (2012–2024). We analyzed nodal metastasis and recurrence by station.

**Results:**

Segmentectomy was more frequent in S6 than in BS. pN1 and pN2 incidence was 8.3% and 2.4% in S6 and 4.3% and 6.8% in BS, respectively. In S6, pN2 metastases were single-station with N1, with no inferior mediastinal involvement. S6 nodal recurrences were confined to #4L/#4R and occurred outside the LND field. In BS, skip pN2 was more frequent and nodal recurrences occurred at #7 within the field.

**Conclusion:**

In clinical stage IA S6 NSCLC, nodal events occurred in the superior mediastinal stations. All pN2 were single-station with N1, and all nodal recurrences occurred after lobe-specific mediastinal LND. Management should follow intraoperative N1 assessment: if negative, S6 segmentectomy without mediastinal LND; if positive, lobectomy with superior mediastinal and subcarinal LND, omitting inferior mediastinal nodes unless specifically suspected.

## Introduction

Anatomic segmentectomy is now an accepted treatment option for small, peripheral non-small cell lung cancer (NSCLC), supported by evidence from randomized studies demonstrating noninferior recurrence-free survival and improved overall survival compared to lobectomy.^1)^ As its indications expand, the oncologic adequacy of limited resection hinges on a precise understanding of segment-specific lymphatic spread patterns and lymph node metastasis (LNM). The superior segment of the lower lobe (S6) is anatomically distinct from the basal segments (BSs). Prior studies involving mediastinal lymph node dissection (LND) indicate that lymphatic spread from segment S6 tends to occur preferentially toward the superior mediastinal lymph nodes (LNs), aligning with the frequent involvement of superior and the rare involvement of inferior mediastinal LNMs.^2–10)^

However, important gaps remain. First, only a few studies have elucidated lymphatic pathways through a combined analysis of pathologic LNM and postoperative isolated nodal recurrence (ILNR), which more directly informs the “missed-node” risk when planning the extent of LND. Second, practical, segment-specific guidance is limited on how to translate these patterns into operative choice and the extent of LND for clinical stage IA S6 NSCLC. Finally, no study has examined whether lymphatic spread patterns within S6 vary by subsegment (S6a/S6b/S6c), which could further inform the oncologic safety and surgical planning of S6 segmentectomy.

To address the above-mentioned gaps, we conducted a single-center retrospective study of clinical stage IA (cT1a–cT1c N0) lower-lobe NSCLC aimed at comparing tumors in the S6 with those in the BS to: (i) delineate station-specific distributions of LNM and ILNR; (ii) assess whether, for S6 tumors, superior mediastinal LND is necessary and whether inferior mediastinal LND can be omitted; and (iii) explore whether subsegmental location within S6 is associated with distinct lymphatic spread patterns. Accordingly, we aimed to distill these patterns into practical, segment-specific recommendations regarding operative choice and the extent of LND in clinical stage IA S6 NSCLC.

## Materials and Methods

### Study design and patients

A total of 2547 patients with NSCLC underwent complete resection via segmentectomy or lobectomy at Aichi Cancer Center Hospital between January 2012 and December 2024. Among these, 932 had tumors located in the lower lobe. Patients were excluded if they met any of the following criteria: (i) clinical stage IB–IVB disease (n = 387); (ii) adenocarcinoma *in situ* or minimally invasive adenocarcinoma (n = 66); (iii) large cell neuroendocrine carcinoma or carcinoid histology (n = 11); and (iv) synchronous or metachronous multiple primary lung cancers (n = 93). Finally, 375 patients with clinical stage IA1–IA3 (cT1a–cT1c N0) NSCLC were included.

**Fig. 1:**
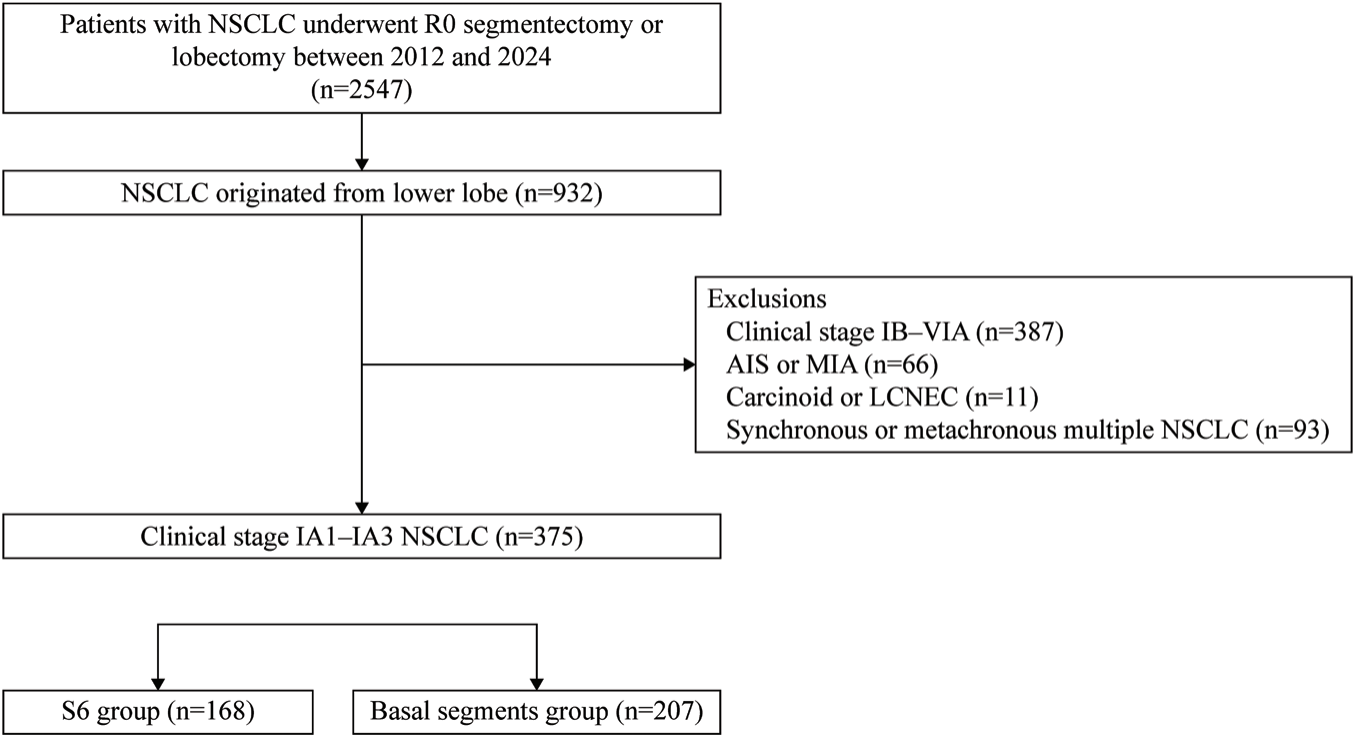
Flowchart of patient selection process. AIS: adenocarcinoma in situ; MIA: minimally invasive adenocarcinoma; LCNEC: large cell neuroendocrine carcinoma; NSCLC: non-small cell lung cancer

Patients were categorized by tumor location into the S6 group (n = 168) and BS group (n = 207) (**Fig. 1**). This study was approved by the Institutional Review Board of Aichi Cancer Center Hospital (IR071026). Owing to the retrospective design, the requirement for written patient consent was waived.

### Imaging and tumor assessment

High-resolution chest computed tomography (CT) (slice thickness, 0.5–1.0 mm) was used to measure tumor size and consolidation. The consolidation-to-tumor ratio (CTR) was defined as the maximum diameter of the solid component divided by the maximum tumor diameter on lung-window images. Radiologic suspicion of LNM was defined as a short-axis diameter >1.0 cm on thin-section chest CT. The tumor-bearing segment was determined using the involved pulmonary vein on preoperative CT. The V6c branch was used as the anatomical landmark separating the S6 from the BS. When the tumor extended into both S6 and BS, the segment with the larger tumor component or containing the draining vein was assigned. Tumors in the S6 were further subclassified into S6a (superior), S6b (lateral), and S6c (medial) subsegments based on the subsegmental pulmonary veins visible on CT. When tumor localization within the segment or subsegment was unclear, the assignment was confirmed on 3-dimensional CT reconstructions generated with REVORAS^TM^ (Ziosoft, Inc., Tokyo, Japan).^11)^

Clinical TNM staging was based on the 8th edition of the TNM Classification of Malignant Tumors. Positron emission tomography-CT (PET-CT) and brain imaging were performed at the treating physician’s discretion.

### Surgical procedure

All patients underwent either lower-lobe segmentectomy or lobectomy with LND. Dissected LNs were classified according to the map defined by the International Association for the Study of Lung Cancer (IASLC).^12)^

N1 LND was performed as follows: in principle, the hilar (#10), interlobar (#11), lobar (#12), and segmental (#13) LNs were dissected during lobectomy or segmentectomy in all patients. During segmentectomy, dissection of #13 in the non-resected segments was not performed. Moreover, given that creating the interlobar fissure is not essential for S6 segmentectomy, dissection of #11L or #11i was often omitted. In this study, stations #10–#11 were defined as hilar/interlobar LNs and #12–#13 as peripheral LNs.

N2 LND was performed either systematically or as a lobe-specific dissection. Systematic mediastinal LND for right lower-lobe tumors included superior mediastinal LNs (#2R, #4R), subcarinal LNs (#7), and inferior mediastinal LNs (#8, #9). For left lower-lobe tumors, aortopulmonary window LNs (#5, #6), left tracheobronchial LNs (#4L), subcarinal LNs (#7), and inferior mediastinal LNs (#8, #9) were included. In contrast, lobe-specific mediastinal LND included only subcarinal and inferior mediastinal LNs.

### Pathological LN evaluation

Pathologic TNM staging was based on the 8th edition of the TNM Classification of Malignant Tumors, and was histologically classified using World Health Organization criteria.^6)^ All dissected LNs were labeled by the IASLC station and reviewed by board-certified pathologists. Skip pN2 metastasis was defined as pN2 without the involvement of any N1 station on pathologic examination of the resection specimen; single-station pN2 as metastasis confined to 1 N2 station, irrespective of N1 status; and multiple-station pN2 as metastasis involving 2 or more distinct N2 stations.

### Postoperative course and follow-up

Adjuvant chemotherapy was recommended for patients with pathologic stage IB (such as tegafur–uracil) or stage II–IIIA disease (such as cisplatin plus vinorelbine). After surgery, patients were followed every 3–6 months for the first 5 years and annually thereafter. Follow-up evaluations included physical examination, chest radiography or CT, and tumor marker testing. When recurrence was suspected, brain magnetic resonance imaging, bone scintigraphy, PET-CT, or biopsy was performed as appropriate. ILNR was defined as the first recurrence event during postoperative surveillance localized to intrathoracic LNs without relapse at any other sites.

### Statistical analysis

Continuous variables are summarized as median (range) and categorical variables as count and percentage. Between-group comparisons were performed using the Mann–Whitney U test. Given the limited number of nodal events, multivariable regression modeling was not performed to avoid model overfitting. Results with 2-sided *P* <0.05 were considered statistically significant. Analyses were performed using EZR (version 1.68; Saitama Medical Center, Jichi Medical University, Saitama, Japan).

## Results

### Patient characteristics

The baseline characteristics of patients in the 2 groups are summarized in **Table 1**. A total of 375 patients were analyzed: 168 in the S6 group and 207 in the BS group. The median age was slightly higher in the S6 group than in the BS group (70 vs. 68 years, *P* = 0.075). Sex distribution, laterality, clinical stage, CTR, and histological type were similar between the groups. Among non-adenocarcinoma tumors, squamous cell carcinoma accounted for 18 of 19 patients in the S6 group and 25 of 27 patients in the BS group, whereas adenosquamous carcinoma accounted for 1 patient in the S6 group and 2 patients in the BS group.

**Table 1:** Baseline patient characteristics

Characteristic	S6 (n = 168)	Basal segments (n = 207)	*P* value
Median age, years (range)	70 (42–90)	68 (36–84)	0.075
Sex (male/female)	80/88	103/104	0.758
Laterality (right/left lung)	100/68	120/87	0.843
Median CTR (range)	1.0 (0.2–1.0)	1.0 (0.3–1.0)	0.149
Clinical stage (IA1/IA2/IA3)	26/93/49	43/102/62	0.344
Surgery (lobectomy/segmentectomy)	108/60	162/45	0.003
LND (hilar/lobe-specific/systematic)	49/83/36	56/98/53	0.639
Histologic type (AD/non-AD)	149/19	180/27	0.726
Pathologic N0/N1/N2	150/14/4	184/9/14	0.051
Isolated LN recurrence	3 (1.7%)	2 (0.9%)	0.680

Non-adenocarcinoma histology included squamous cell carcinoma (18/19 S6 group patients; 25/27 BS group patients) and adenosquamous carcinoma (1/19 S6 group patients; 2/27 BS group patients).

CTR: consolidation-to-tumor ratio; LND: lymph node dissection; AD: adenocarcinoma; BS: basal segment

The proportion of patients who underwent segmentectomy was significantly higher in the S6 group than in the BS group (35.7% vs. 21.7%, *P* = 0.003). The extent of LND did not differ significantly between the groups (*P* = 0.639). Regarding pathological nodal status, there was no significant difference in the overall incidence of LNM: pN1 was observed in 14 patients (8.3%) in the S6 group and 9 patients (4.3%) in the BS group (*P* = 0.131), whereas pN2 was detected in 4 patients (2.4%) in the S6 group and 14 patients (6.8%) in the BS group (*P* = 0.054). Postoperative recurrence occurred in 16 patients (9.5%) in the S6 group and 15 patients (7.2%) in the BS group. ILNR was rare in both groups (1.7% vs. 0.9%, *P* = 0.68).

### Clinical factors associated with occult LN metastasis

**Fig. 2:**
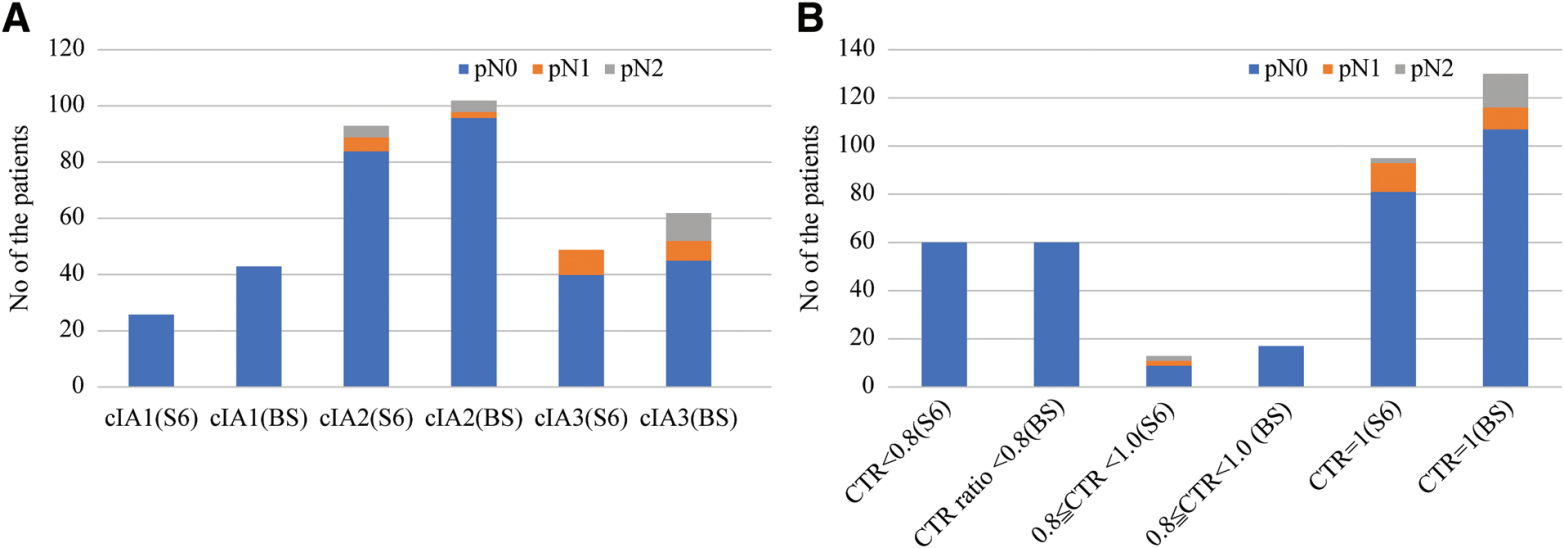
Association between clinical factors and occult lymph node metastasis in clinical stage IA lower-lobe NSCLC. Stacked bar charts showing the proportions of pN0, pN1, and pN2 among tumors arising in the S6 and BS across clinical stages (**A**) and the CTR (**B**). NSCLC: non-small cell lung cancer; BS: basal segments; CTR: consolidation-to-tumor ratio

We investigated the clinical factors associated with occult LNM in clinical stage IA NSCLC in the S6 and BS groups. Notably, in our study, all LNM events occurred in patients with clinical stage IA2 or IA3 tumors and not in those with stage IA1 tumors (**Fig. 2A**). We did not observe LNM in tumors with CTR <0.8 (**Fig. 2B**).

### Occult nodal metastases by station

**Fig. 3:**
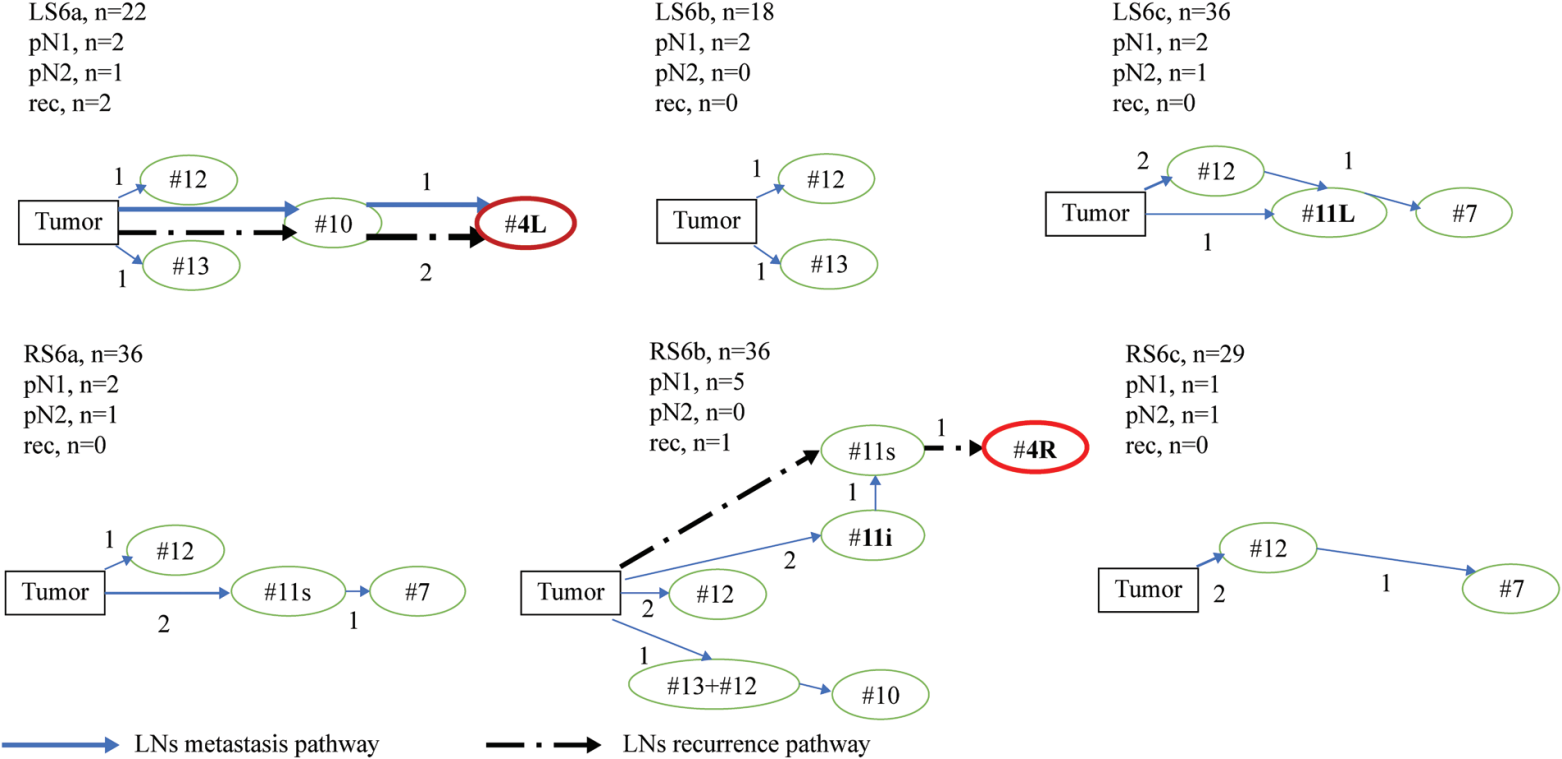
Summary of lymph node metastasis and recurrence pathways in the S6 group. Schematic summary of LN pathways within the S6 segment at the subsegment level. Arrows depict observed routes from the primary lesion to nodal stations. Solid lines indicate pathological LN metastasis (pN1/pN2); dashed lines indicate isolated nodal recurrence after curative resection. Line thickness reflects the observed frequency within each subsegment. L: left; R: right; LN: lymph nodes; rec: lymph node recurrence

To characterize the patterns of lymphatic spread in S6 tumors, we examined the distribution of occult LNM (**Table 2**). In the S6 group, 14 patients had pN1: 5 (36%) had metastases in hilar/interlobar LNs (#10, #11), 8 (57%) in peripheral LNs (#12, #13), and 1 (7%) in both stations (**Fig. 3**). In contrast, in the BS group, 9 patients had pN1, with 3 (33%) presenting metastasis in hilar/interlobar LNs and 6 (67%) in peripheral LNs. All 4 patients in the S6 group with pN2 had single-station pN2, and none demonstrated skip pN2. Notably, 1 patient presented metastasis to a superior mediastinal LN (#4L).

**Table 2:** Patterns and frequencies of occult lymph node metastasis in clinical stage IA NSCLC: the S6 versus Basal segments groups

	S6	Basal segments
pN1	N = 14	N = 9
Hilar/interlobar LNs (#10, #11)	5 (36%)	3 (33%)
Peripheral LNs (#12, #13)	8 (57%)	6 (67%)
Hilar/interlobar and peripheral LNs	1 (7%)	0
pN2	N = 4	N = 14
Skip N2 (#4R, #4L)	0	0
Skip N2 (#7)	0	4 (29%)
Skip N2 (#8, #9)	0	1 (7%)
Single-station N2 (#4L)	1 (25%)	0
Single-station N2 (#7)	3 (75%)	5 (36%)
Single-station N2 (#8, #9)	0	1 (7%)
Multiple-station N2 (#4L+#7)	0	2 (14%)
Multiple-station N2 (#7+#8, #9)	0	1 (7%)

LNs: lymph nodes; NSCLC: non-small cell lung cancer

These findings contrasted with the trend observed in BS group patients, who frequently exhibited skip pN2 metastasis (n = 5) involving the subcarinal LN (#7, n = 4) or the inferior mediastinal LNs (#8/9, n = 1). Superior mediastinal metastasis (#4L) was observed in 2 patients in the BS group, both with multiple-station pN2.

### Postoperative nodal recurrence patterns

Given that some nodal recurrences were confined to non-dissected stations and might have been identified as pN1 or pN2 if the LND had extended beyond the actually dissected field, we analyzed station-specific postoperative nodal recurrence patterns (**Table 3**). All ILNR events occurred in patients with clinical stage IA3 tumors and a CTR of 1. In the S6 group, 3 patients showed ILNR. All patients had pN0 or pN1 disease at the time of lobectomy with lobe-specific mediastinal LND and experienced recurrence in the superior mediastinal LNs (#4L or #4R), which were outside the LND field. In contrast, 2 patients in the BS group showed ILNR, both of whom had pN2 disease at surgery. These ILNR events occurred at the subcarinal LN (#7), which was within the LND field. Generally, these findings suggest that IA2–IA3 NSCLC in S6 may metastasize to the superior mediastinal or subcarinal LNs via N1 nodes, whereas metastasis to the inferior mediastinal LNs is extremely rare.

**Table 3:** Details of cases with isolated LN recurrence in the S6 and basal segments groups

Case	Segment	cStage	CTR	pN	Surgery	Recurrence	
						N1	N2
S6-1	S6a	IA3	1.0	N1 (#13)	LLL + lobe-specific	—	#4L
S6-2	S6a	IA3	1.0	N0	LLL + lobe-specific	#10	#4L
S6-3	S6b	IA3	1.0	N1 (#11s)	RLL + lobe-specific	#11s	#4R
BS-1	S10	IA3	1.0	N2 (#11, #7)	RLL + systematic	#10	#7
BS-2	S10	IA3	1.0	N2 (#11s, #7)	LLL + lobe-specific	—	#7

All listed recurrences were confirmed pathologically or by CT or PET-CT.

CTR: consolidation‑to‑tumor ratio; pN: pathologic nodal category; LLL: left lower lobectomy; RLL: right lower lobectomy; Lobe-specific: lobe-specific mediastinal lymph node dissection; Systematic: systematic mediastinal lymph node dissection

### Distribution of LN metastasis and recurrence by subsegmental location of S6 tumors

To address any trend in LNM by tumor location at the subsegmental level, we further subclassified S6 tumors as follows: left S6a (n = 22), left S6b (n = 18), left S6c (n = 27), right S6a (n = 36), right S6b (n = 36), and right S6c (n = 29) (**Fig. 3**). Notably, superior mediastinal LNM or ILNR occurred in left S6a and right S6b. Left S6a had 1 metastasis to #4L and 2 recurrences at #4L, whereas right S6b had 1 recurrence at #4R. No such events were identified in S6c tumors. These findings suggest that lymphatic spread patterns may differ at the subsegmental level; recognizing such patterns may help refine surgical strategy by optimizing the extent of mediastinal LND, although confirmation in larger studies is warranted.

## Discussion

In this study, clinical stage IA S6 NSCLC exhibited distinct patterns of LNM and recurrence compared to tumors originating from the BS. In particular, pN1 metastasis in the S6 group occasionally involved interlobar LNs such as #11i and #11L, which are often outside the standard N1 LND field during S6 segmentectomy. This raises concern about pathologic understaging if these LNs are not adequately assessed, despite negative findings in other N1 stations. Therefore, intraoperative suspicion of these LNs should prompt either conversion to lobectomy or targeted sampling with intraoperative pathologic assessment to ensure accurate staging. Yoshimura et al. reported that, among S6 tumors ≤20 mm, 38.9% of nodal metastases involved interlobar LNs compared with 11.1% in BS tumors, underscoring the oncologic importance of these stations^5)^ (**Supplementary Table 1**). Ichinose et al. revealed that skip pN1 metastasis to interlobar nodes was more frequent in lower-lobe tumors, especially in the S6 group, and that pleural invasion was associated with a higher rate of such metastasis, although their study included stage II or higher cases.^6)^

Regarding pN2, all patients in the S6 group with pN2 had single-station pN2 without skip pN2. In 1 patient, metastasis to the superior mediastinal LNs (#4L) was observed. This segment-specific lymphatic spread pattern is consistent with the findings of Watanabe et al., who reported that 20% of S6 tumors metastasized to superior mediastinal LNs, compared with only 4% in the BS group.^7)^ Yuhara et al. reported that among patients with pure-solid NSCLC ≤5 cm, superior mediastinal LNM occurred exclusively in the S6 group and not in the BS group.^8)^

In our study, all isolated LN recurrences in the S6 group were observed exclusively in superior mediastinal LNs (#4L or #4R), including patients with pN0 or pN1 disease at the time of lobectomy with lobe-specific mediastinal LND. These recurrence sites were outside the lobe-specific LND field. Moreover, a supplemental analysis of the randomized JCOG0802/WJOG4607L trial showed that mediastinal LN recurrence was significantly more frequent in patients with S6 tumors who underwent lobe-specific LND than in those who underwent systematic LND.^9)^ These findings support an anatomically rational and oncologically appropriate approach to LND, with particular attention to superior mediastinal LNs in S6 tumors—even when classified as clinical stage IA.

In contrast, metastasis and recurrence involving inferior mediastinal LNs (#8, #9) were absent in all patients in the S6 group in our study. This finding suggests that dissection of these LNs may be omitted in clinical stage IA S6 tumors unless radiologic or intraoperative findings indicate otherwise. Similarly, Maniwa et al. reported that inferior mediastinal LNM occurred only in the BS group, whereas patients in the S6 group virtually had no metastasis to #8 or #9.^10)^

At a finer anatomical level, our findings suggest that lymphatic behavior within the S6 may vary by subsegment. Only tumors located in S6a and S6b—but not in S6c—were associated with superior mediastinal LN metastasis or recurrence. To our knowledge, no previous study has reported such intrasegmental variations in lymphatic spread within the S6. These findings suggest inherent anatomical differences in bronchovascular or lymphatic architecture between subsegments and are unlikely to be explained by tumor size, histologic type, or clinical stage alone. Although these aspects were not the primary focus of our study and were based on a limited number of patients, the observations raise a novel anatomical consideration that may have implications for the oncologic safety of S6 segmentectomy.

Previous studies on prognostic differences between S6 and BS tumors have yielded inconsistent results. Lomangino et al. reported that tumors in the S6 group were associated with poorer outcomes than those in the BS group (5-year disease-free survival, 58.3% vs. 81.5%), likely due to a higher incidence of locoregional recurrence in the S6 group (41.7% vs. 15.4%).^13)^ In contrast, a meta-analysis by Yu et al., involving 1727 patients from 5 studies, found no significant difference in 5-year overall survival between the groups (hazard ratio, 0.91; 95% confidence interval, 0.68–1.22).^14)^ These conflicting results may reflect differences in surgical practice, LND strategies, and patient backgrounds across studies.

Collectively, our findings underscore the importance of understanding segment- and subsegment-specific lymphatic spread when selecting the surgical procedure—whether lobectomy or segmentectomy—and determining the extent of LND. In particular, careful assessment of interlobar and superior mediastinal LNs is essential in S6 tumors, whereas dissection of inferior mediastinal LNs may be reasonably omitted when no abnormalities are found on imaging or during surgery. For clinical stage IA S6 NSCLC—particularly solid-dominant lesions—our findings support a strategy that ensures rigorous evaluation of N1 station LNs with special attention to #11i/#11L, prioritizes assessment of superior mediastinal stations, and recognizes that routine inferior mediastinal LND may be unnecessary in the absence of specific suspicion.

This study has some limitations. First, it was conducted retrospectively at a single institution, which may limit generalizability. Second, the extent of LND—especially in segmentectomy cases—was not fully standardized, potentially affecting nodal assessment. Third, subsegmental analysis within S6 was limited by a small cohort, and intraoperative evaluation of interlobar LNs (#11L and #11i) was not consistently documented. Fourth, we did not perform a separate analysis of LN metastasis according to histologic subtype because the proportion of non-adenocarcinoma tumors was small and similar between the S6 and BS groups, and such an analysis would have been underpowered. Finally, variation in follow-up duration over the long study period may have affected recurrence detection.

## Conclusion

In clinical stage IA S6 NSCLC, nodal events—metastasis or ILNR—were concentrated at superior mediastinal stations; no inferior mediastinal metastasis or recurrence was observed. All pN2 cases were single-station and accompanied by N1 metastasis. Furthermore, all postoperative nodal recurrences occurred in patients with either N1 metastasis or N1-station recurrence, and all patients had undergone lobe-specific mediastinal LND that excluded superior mediastinal nodes, indicating that the recurrences arose outside the dissected field. Based on these findings, we propose a strategy guided by intraoperative pathologic assessment of N1 nodes: if no N1 metastasis is found, S6 segmentectomy without mediastinal LND should be performed; if N1 metastasis is present, lobectomy with dissection of the superior mediastinal and subcarinal nodes should be performed while omitting inferior mediastinal nodes (#8/#9). When stratified by S6 subsegment, nodal metastases and ILNRs were observed in S6a and S6b, but none in S6c; these subsegment-specific differences require further research.

## Supplementary Materials

Supplementary Table 1

## Declarations

### Data availability

The data that support the findings of this study are available from the corresponding author on reasonable request.
