# Supplementary material for: Lymph Node Metastasis and Recurrence Patterns in Clinical Stage IA Lower-Lobe Non-Small Cell Lung Cancer: Toward an Optimal Surgical Strategy for Superior Segment (S6) Tumors
Source: Ann Thorac Cardiovasc Surg. 2026 Jan 14;32(1):25-00198. doi: 10.5761/atcs.oa.25-00198 (PMC12812425; doi:10.5761/atcs.oa.25-00198)
Supplement: Supplementary Table 1 [file atcs-32-1-25-00198-s001.pdf]

Supplementary Table 1. Summary of superior versus inferior mediastinal LN metastasis and isolated nodal recurrence in clinical stage I S6 NSCLC

Report	Clinical stage	Number of patients	Surgery	pN1 /pN2 n(%)	Lymph node metastasis<br>Superior/Inferior LNs n(%)	Isolated nodal recurrence<br>Superior/Inferior LNs n(%)
Handa et al 2017〔4〕.	cIA-IB	60	Segmentectomy or Lobectomy + lobe-specific or systematic LND	4(6.6%)/ 9(15%)	-	4(6.6%)/ 0
Yoshimura et al 2022〔5〕.	cIA2	51	Segmentectomy or Lobectomy + systemic LND	6(11.8%)/ 3(5.9%)	1(1.9%)/ 0	-
Maniwa et al 2024〔9〕.	cIA1-IA2	196	Segmentectomy or Lobectomy + lobe-specific or systematic LND	7(3.5%)	1(1.0%)/ 0	-
This study	cIA1-IA3	168	Segmentectomy or Lobectomy + hilar or lobe-specific or systematic LND	14(8.3%)/ 4(2.3%)	1(0.6%)/ 0	3(1.7%)/ 0

Abbreviations: LNs, lymph nodes; LND, lymph node dissection.
